# Frequency and variability of dental morphology in deciduous and permanent dentition of a Nasa indigenous group in the municipality of Morales, Cauca, Colombia

**Published:** 2014-03-30

**Authors:** Eider Díaz, Lorena García, Michelle Hernández, Lesly Palacio, Diana Ruiz, Nataly Velandia, Judy Villavicencio, Freddy Moreno

**Affiliations:** 1 School of Dentistry at the Universidad del Valle. Cali, Colombia.; 2 School of Basic Sciences at the Universidad del Valle. Cali, Colombia.; 3 School of Health Sciences at the Pontificia Universidad Javeriana. Cali, Colombia.

**Keywords:** Dental anthropology, dental morphology, non-metric dental traits, indigenous Nasa

## Abstract

**Objectives:**

To determine the frequency, variability, sexual dimorphism and bilateral symmetry of fourteen dental crown traits in the deciduous and permanent dentition of 60 dental models (35 women and 25 men) obtained from a native, indigenous group of Nasa school children of the Musse Ukue group in the municipality of Morales, Department of Cauca, Colombia.

**Methods:**

This is a quantitative, descriptive, cross-sectional study that characterizes dental morphology by means of the systems for temporary dentition from Dahlberg (winging), and ASUDAS (crowding, reduction of hypocone, metaconule and cusp 6), Hanihara (central and lateral incisors in shovel-shape and cusp 7), Sciulli (double bit, layered fold protostylid, cusp pattern and cusp number) and Grine (Carabelli trait); and in permanent dentition from ASUDAS (Winging, crowding, central and lateral incisors in shovel-shape and double shovel-shape, Carabelli trait, hypocone reduction, metaconule, cusp pattern, cusp number, layered fold protostylid, cusp 6 and cusp 7).

**Results:**

The most frequent dental crown features were the shovel-shaped form, grooved and fossa forms of the Carabelli trait, metaconule, cusp pattern Y6, layered fold, protostylid (point P) and cusp 6. Sexual dimorphism was not observed and there was bilateral symmetry in the expression of these features.

**Conclusions:**

The sample studied presented a great affinity with ethnic groups belonging to the Mongoloid Dental Complex due to the frequency (expression) and variability (gradation) of the tooth crown traits, upper incisors, the Carabelli trait, the protostylid, cusp 6 and cusp 7. The influence of the Caucasoide Dental Complex associated with ethno-historical processes cannot be ruled out.

## Abstract (es)

**Objetivos:**

Determinar la frecuencia, la variabilidad, el dimorfismo sexual y la simetría bilateral de 14 Rasgos Morfológicos Dentales Coronales (RMDC) en dentición temporal y permanente de 60 modelos (35 mujeres y 25 hombres) obtenidos de un grupo de indígenas escolares Nasa del cabildo Musse Ukue del municipio de Morales, departamento del Cauca.

**Métodos:**

Estudio descriptivo transversal cuantitativo que caracteriza la morfología dental mediante los sistemas en dentición temporal Dahlberg (winging), Asudas (crowding, reducción de hipocono, metacónulo y cúspide 6), Hanihara (incisivos centrales y laterales en pala y cúspide 7), Sciulli (doble pala, protostílido, pliegue acodado, patrón cuspídeo y número de cúspides) y Grine (cúspide de Carabelli); y en dentición permanente Asudas (Winging, crowding, incisivos centrales y laterales en pala, doble pala, cúspide de Carabelli, reducción del hipocono, metacónulo, patrón cuspídeo, número de cúspides, pliegue acodado, protostílido, cúspide 6 y cúspide 7).

**Resultados:**

Los RMDC más frecuentes fueron la forma de pala, formas surco y fosa de la cúspide de Carabelli, metacónulo, patrón cuspídeo Y6, pliegue acodado, protostílido (punto P) y cúspide 6. No se observó dimorfismo sexual y hubo simetría bilateral en la expresión de los rasgos.

**Conclusiones:**

La muestra estudiada presenta una alta afinidad con grupos étnicos pertenecientes al Complejo Dental Mongoloide debido a la frecuencia (expresión) y variabilidad (gradación) de los RMDC, incisivos en pala, la cúspide Carabelli, el Protostílido, la cúspide 6 y la cúspide 7; sin embargo, no se puede descartar influencia del Complejo Dental Caucasoide asociado con procesos etno-históricos.

##  Introduction 

Analysis of dental morphology in the context of dental anthropology seeks to understand the manner in which the frequency, sexual dimorphism and bilateral symmetry of Tooth Crown Morphological Traits (TCMT) present in deciduous and permanent teeth 1. The TCMT consist of phenotypic enamel forms expressed and regulated by the genome of an individual and population during odontogenesis. Structures can be positive (tubercular) or negative (inter-tuberculars and fosomorfos) that have the potential to be present, or not, at a specific site (frequency) and in a different form (variability) in one or more members of a population group 2 ^-^ 6.

Based on the TCMT, dental morphology is studied from an interdisciplinary viewpoint (biology, anthropology, dentistry, paleopathology, archeology, forensic science) because teeth can be used in the estimation of biological relationships between populations. This is accomplished by comparatively analyzing past and present human groups in an attempt to clarify the historical, cultural and biological macro and micro-evolutionary processes that lead to an understanding of the origin, formation, contacts, displacements, migrations pathways and isolates that have led to the populating of the planet and ethnic variation of humanity 1 ^,^ 3 ^,^ 7 ^-^ 8. It similarly constitutes an accurate means of recognizing individuals whose death makes it difficult to distinguish them by other processes which are part of the individual reconstruction of osteobiography (odontography) or for that of the general population 1 ^,^ 8 ^-^ 9.

This research seeks to observe the expression of 14 TCMT´s from a group of Nasa indigenous people from the Musse Ukue council of in the municipality of Morales, province of Cauca, for the purpose of further processing of dental morphological markers that contribute to the ethnographic description of Southwestern Colombia. This is an essential process in forensic dental identification to document the elements of the basic identification quatrain (sex, age, ethnicity and height) 9.

## Materials and Methods

###  Population and sampling 

This is a quantitative, cross-sectional, descriptive study of the frequency, variability, sexual dimorphism and bilateral symmetry of 14 TCMT´s in deciduous and permanent dentition from study models obtained from a group of school children of the indigenous Nasa tribe of the Musse Ukue council of the municipality of Morales, Cauca, Colombia. The school children were from Colombian Nasa parents and grandparents and were between the ages of 5 to 18 years. Students were selected from a survey and interview examination who met the inclusion criteria (with clinically healthy teeth where the TCMT were taken into account) and who were asked to sign an informed consent and had parents that authorized their participation via an informed consent agreement. The sample number was selected by convenience based on the central limit theorem which considers a sample of 30 to be significant because "regardless of the functional form of the population from which the sample is drawn, the distribution of sample means calculated with samples of size n drawn from a population with a mean m and finite variance s^2^, approaches a normal distribution with mean m and standard error s^2^/n, when n increases. If the n is large, n≥ 30, the distribution of sample means can approximate a normal distribution" 10.

### Standardization of morphological analysis

The six observers learned to manage the odontoscopic systems (Asudas-*Arizona State University Dental Anthropology System*-, Dahlberg, Hanihara, Sciulli and Grine) with a standardization protocol and double blind in nature to control bias and achieve a unified observational criteria, according to that described by Nichol and Turner II 11.

### Observation

Once the research gained the support of the Research Ethics Committee for Human Subjects of the School of Health, Universidad del Valle and in accordance with Article 11 of Resolution 008430 of the Ministry of Social Protection 12 and with the Helsinki Declaration 13, who classified this study as being without risk, we then proceeded to the observation of 14 TCMT in plaster models using 10-power magnification from a Hu-Friedy^®^ fine point explorer and from gradations of Dahlberg (winging) deciduous dentition system, Asudas (crowding, reduced hypocone, metaconules and cuspids 6) , Hanihara (central and lateral incisors in shovel-shape and cusp 7), Sciulli (double shovel-shaped, protostylid, layered fold, groove pattern and number of cusps) and Grine (Carabelli cusp); and in permanent dentition through Asudas (Winging, crowding, central and lateral incisors in shovel-shaped , double shovel-shaped, Carabelli cusp, hypocone reduction, metaconules, groove pattern, number of cusps, layered fold, protostylid , cusp 6 and cusp 7) (see Table 1 ).

**Table 1.**
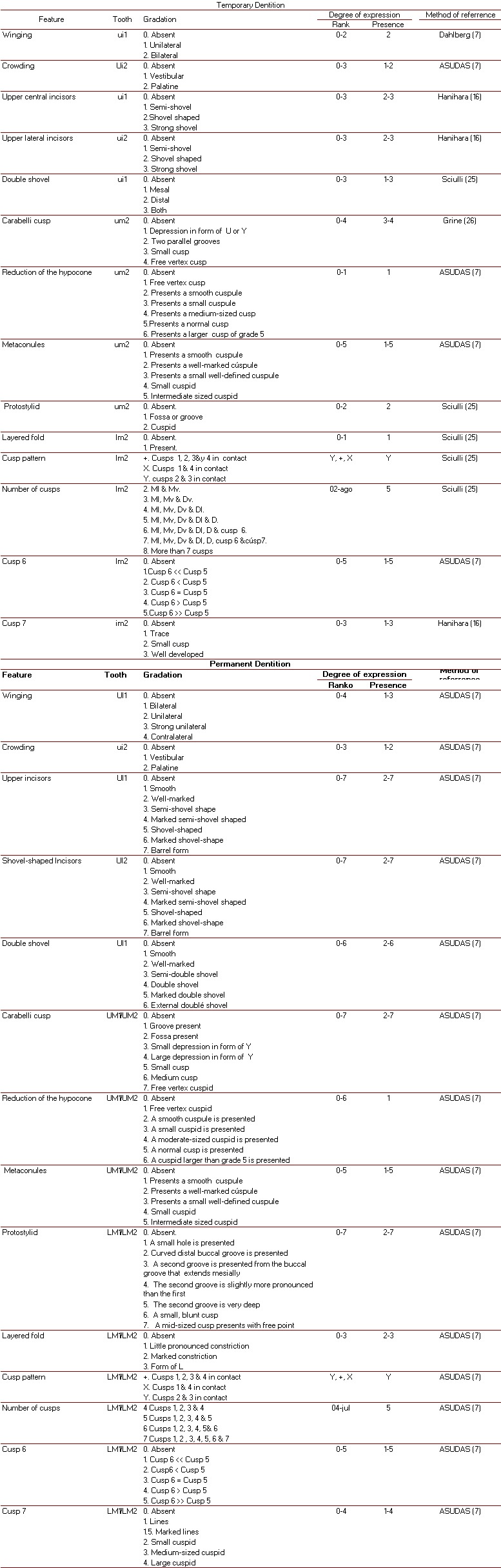
Methods of observation of MCDT

### Statistical Analysis

The data obtained from TCMT observation were entered into a template on Excel^®^ and processed with the SPSS^®^, 17.0 Software through descriptive tests and by univariate and bivariate analysis tests using nonparametric tests (Wilcoxon and Mann-Whitney U) for each of the morphological features. A probability level of <0.05 was considered statistically significant. To determine biological distances the distance matrix was used starting with the hierarchical cluster classification by means of the Euclidean distance squared, while the dendrogram was obtained by using the Ward method.

## Results

Estimating the degree of concordance for standardization of observers was done by means of the Kappa test with Stata^®^ 6.0 Software. Criteria for acceptable inter-observer reliability (observer vs. rater) were 93%, 80% , 85%, 92%, 92% and 87% for the five observers; and intra-observer (observer vs. observer) were 90%, 81%, 81%, 81%, 87% and 85%, respectively.

**Figure 1.**
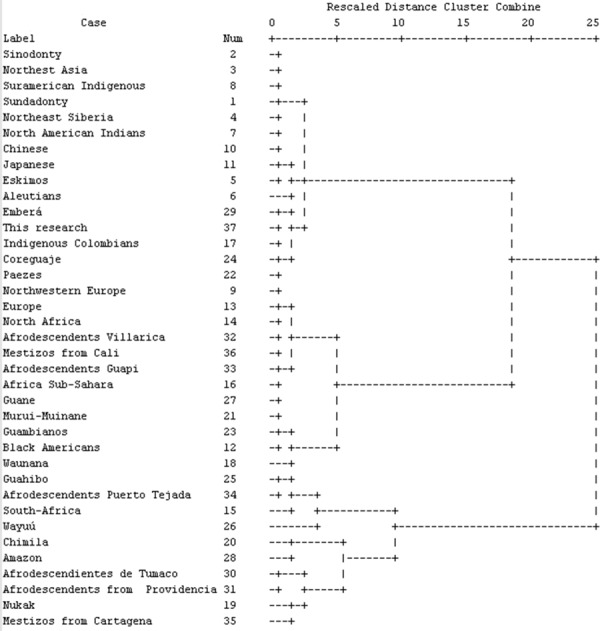
Dendrogram obtained from the distance matrix among world populations based on squared Euclidean distance.

Frequencies were obtained for each of the TCMTs for the 60 models studied (35 females and 25 males). The most common features were the shovel-shaped central and lateral incisors, high frequencies of the groove and fossa forms of Carabelli´s cusp, the metaconules, the Y6 cusp pattern, layered fold, protostylid and cusp 6. None of the features presented sexual dimorphism (*p* <0.05) except for the upper incisors in the deciduous teeth, and all TCMTs are expressed bilaterally (*p* <0.05) (see Table 2). With respect to biological distance in the distance matrix (Table 3) as well as in their respective dendrograms (Fig. 1) it can be seen that the sample has a high affinity for the Mongoloid Dental Complex due to the frequency of the TCMT incisors with shovel shape, Carabelli´s cusp, the protostylid, the cusp 6 and cusp 7. The distance matrix has a sensitivity (Bootstrapping - 1000 resampling method) of *p*= 0.49.

**Table 2.**
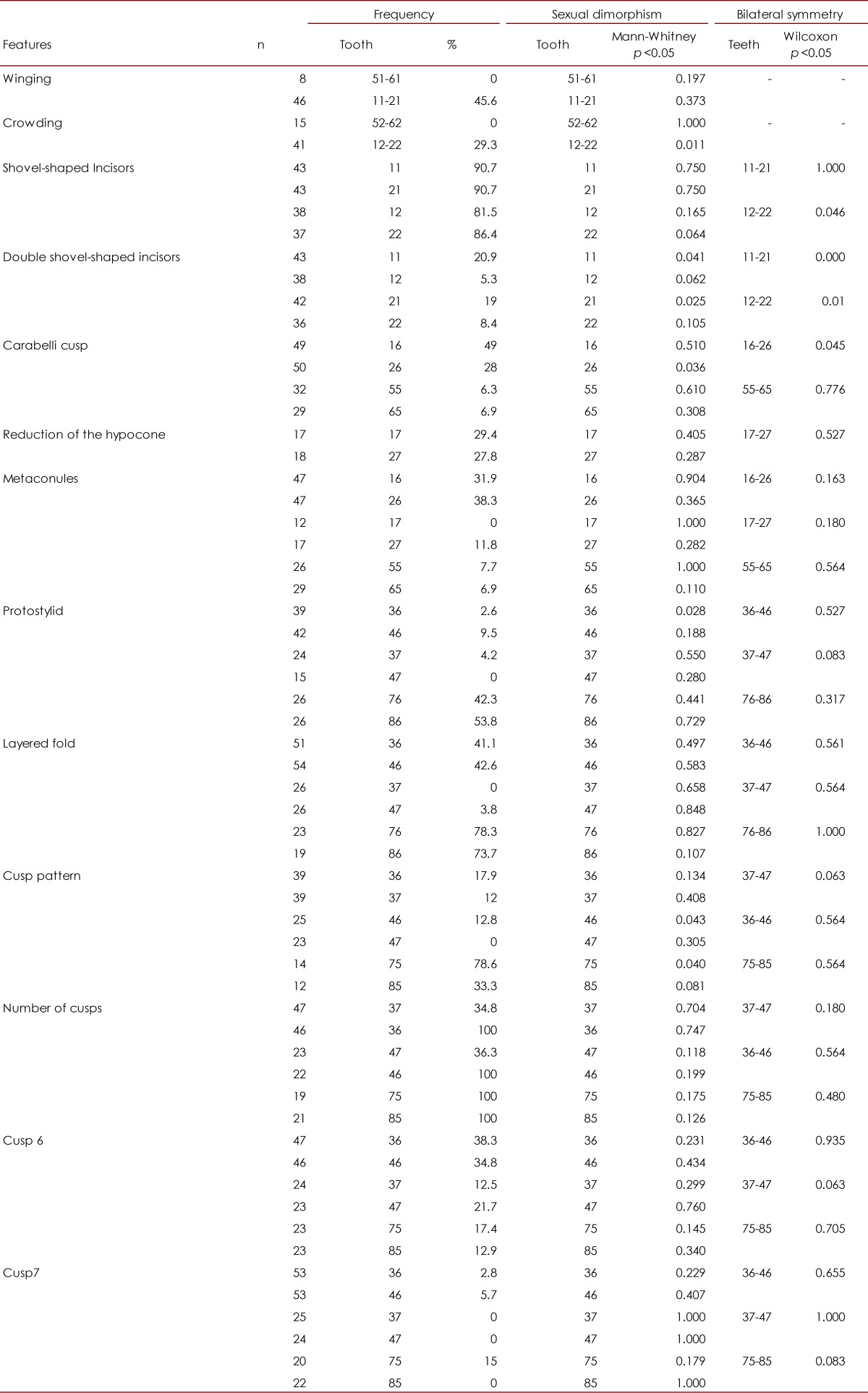
Relative frequencies of dental morphological features

**Table 3.**
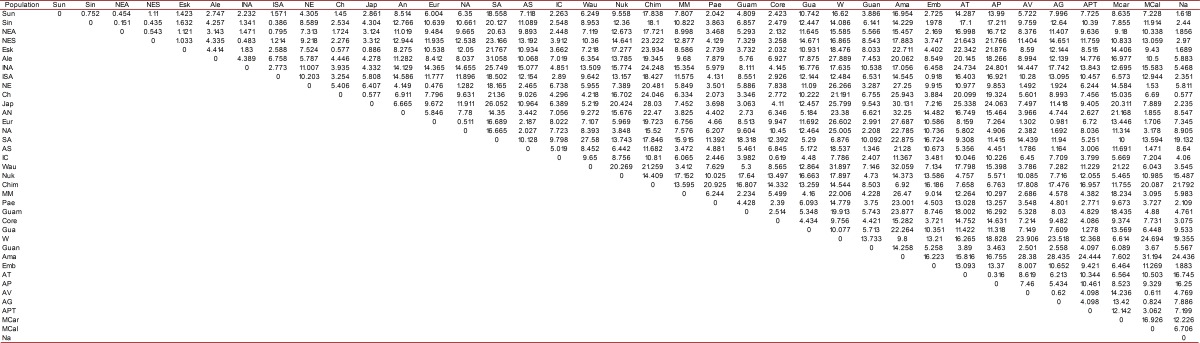
Distance matrix among world populations based on Euclidean squared distances

## Discussion

###  Dental crown morphological traits Winging, Crowding, central and lateral incisors in shovel and double shovel shape 

*Winging* involves rotating one or both upper central incisors relative to the midline. Scott and Turner 7 relate this feature with the absence of space in the dental arch that prevents the proper alignment of the incisors. According to Rodríguez 8, the meso-lingual rotation of both incisors is considered the product of genetic factors characteristic of native American populations while the rotation of a single tooth or both in a disto-lingual direction is caused by crowding. In a population of Amazonian Indians this feature was observed with high frequency 14. In this study for the deciduous dentition *winging* was absent, while in permanent dentition a relative frequency of the trait was observed, represented in grade 1 expression (disto-buccal rotation of both incisors). There was no sexual dimorphism.

Regarding *crowding* or overcrowding of the upper lateral incisors, it is more common in Mongoloid populations and less frequent in Caucasian populations 9, but it was absent in temporary dentition and was low in frequency with permanent dentition. Sexual dimorphism was associated with an increased number of females.

The shovel shape showed a high frequency, mainly in the middle grades (semi-shovel and marked semi-shovel). Also, bilateral symmetry was evident along with the absence of sexual dimorphism. Turner 14 observed this trait in 98.8% of East Asians and 99.8% of South American Indians. Among indigenous people from Colombia, it varies between 50% and 100% 9. Aragón *et al,* 14. observed high frequencies among indigenous tribes in the Amazon basin.

In the case of the double-shovel form, its low frequency is highlighted (smooth grades and lines), bilateral symmetry and sexual dimorphism associated with a greater number of females. Aragón et al 14. also observed high frequencies in Amazonian indigenous groups.

### Carabelli´s cusp, reduction of the hypocone and metaconules

The cusp or tubercle of Carabelli was considered virtually absent from the sample. However, it is important to note that in both dentitions the groove and fossa shapes predominated over the cusp forms with dichotomous expression, which is characteristic of American indigenous peoples 9. Hanihara 16 observed low frequencies of this trait among Japanese and high averages in Blacks and White Americans and found that it distinguishes Caucasians from Asian populations. In the latter case it is predominately in the groove and fossa shapes. Turner 15 found significant expressions among sinodonts, South American Indians, and Northeast Europeans. Among indigenous Colombians, the frequency varies between 20% and 90% 9 and the groove and fossa shapes predominate over the cusp form 4.

Severe reduction and the absence of the disto-lingual cusp (hypocone) is a valued trend from the first upper molar to the second upper molar 9 and is associated with a simplification of the dental morphology and reduced size. In this study, the reduction of the hypocone resulted in the absence of the bilateral form in both sexes. Hypocone is most prevalent in grades 0 and 1. In indigenous Colombians, it is between 80% and 100%. The global variation is between 13% and 95%, with a minimum found among Australian aborigines and a maximum among Mongoloids, which was true in regard to this study 9.

Regarding metaconules, or small cuspid with a free tip located at the distal edge of the upper molars between the disto-buccal and disto-palatal cuspids, they were absent from the sample. This is consistent with that reported in the literature since in American populations, whether prehispanic indigenous or contemporary, the trend is toward decreasing expression 7.

### Protostylid and layered fold

The frequency of this feature in deciduous dentition was significant from the expression of thin cusps with blunted tips on the labial surface of the meso-buccal cusp of the lower second molars. In permanent teeth this trait was practically absent, but significant expression was found in grade 1 (point P or *foramen caecum*) in permanent teeth, which has been defined as an americanoide trait. There was no sexual dimorphism; however there was bilateral symmetry 7 ^,^ 9 ^,^ 17.

Concerning the layered fold, which describes the way in which the meso-lingual cusp is directed toward the central fossa of the temporary and permanent lower molars, a high frequency in both dentitions is reported with bilateral symmetry and no sexual dimorphism. The TCMT is an important marker in sinodont populations, and with a high frequency in indigenous Colombians between 60% and 100% 9.

### Groove pattern and number of cusps

The groove pattern of the second lower deciduous molars and first lower permanent molars describes the configuration of contact of the cusps and their number. The classic pattern is "Y", or "Driopitecino" originating from past Asian populations, along with ¨X¨ configurations and the "+" or "cruciform" which are all considered as reductions that are frequently observed in Caucasoid groups 9. Among Colombian indigenous populations a predominance of Y6 and Y7 configuration is reported (temporary dentition) wherein the Y cusp pattern has a strong genetic control that allows it to be retained from the last Asian populations that inhabited the Americas by way of the Bering land bridge from which prehispanic and current Amerindian populations are derived 14. In this study, in temporary dentition the most frequent cusp pattern is Y5, while in permanent dentition, the first molar is +5 and the second molars are +4 which may be associated with miscegenation. This trait does not present with sexual dimorphism and had bilateral expression.

### Cusp 6 and 7

The frequency and variability of cusp 6 (located between the disto-buccal and disto-lingual cusps of the lower molars) and cusp 7 (located on the marginal edge between the meso-lingual and disto-lingual cusps of the lower molars) are present in both dentitions. They are, not considered to be at significant levels if you consider that cusp 6 is characteristic of Asian populations and cusp 7 of Negro populations 14.

### Biological distance and dental complexes

Because of the frequency and variability of TCMTs, human populations can be associated with geographical distributions, and different researchers have ethnographically classified human beings in complex populations from dental morphology. The first of these complexes was defined by Hanihara in 1966 16 as the Mongoloid Dental Complex, which brings together different populations from East Asia that are characterized by having a complex dental morphology represented in a high frequency of shovel-shaped, layered fold and cusp pattern 6 incisors. Later, Turner in 1984 15 divided the Mongoloid Dental Complex into two groups. The first subdivision, or Sinodont, composed of Northeast Asian populations, is characterized by the addition and enhancement of some TCMTs, such as the shovel-shaped and layered fold incisors, along with the Y6 cusp pattern, the protostylid (cusp forms) and *winging*. The second subdivision or sundadont covers Southeast Asian populations that have retained an ancestral condition and have simplified the expression of some morphological traits.

On the other hand, Zoubov 16 proposed a dental delimitation of global populations into two complexes: the Eastern Dental Complex, the equivalent of the Mongoloid Dental Complex proposed by Hanihara; and the Western Dental Complex, consisting of Northern Caucasoid and Negroid populations (Southern Caucasoid populations) characterized by the high frequency of the Carabelli cusp (cusp forms), of the cusp pattern X, the groove pattern + and the cusp 7 more prevalent in Negroid populations. Irish 18 subdivided the Negroid populations of Southern Africa (Western Dental Complex) into the Sub-Saharan Dental Complex and North African Dental Complex.

Edgar (2007) 19 grouped humans into five clusters: the Mongoloid Dental Complex formed by sinodont and sundadont groups; the Caucasoid Dental Complex formed by Western Eurasian groups (Europe, North Africa, Middle East and India); the Saharan African Dental Complex (composed of West African and South African subgroups closer to sundadont populations of the South Pacific), several Pacific groups of Sahul or Oceania and American Paleo-Indians who present frequencies and morphological variations that take them out of the complexes described.

In the case of the American populations, currently the model proposed by Turner in 1984 15 is accepted. It suggests that the populating of the American continent initially occurred by human sinodonts who migrated from Northern China and crossed Bering land bridge. This is affirmed by past and present American indigenous groups having a sinodont dental morphology, and therefore, should be included in the Mongoloid Dental Complex, which is in agreement with the miscegenation with other ethnic groups. The high frequency of shovel-shaped, layered fold incisors, protostylid and cusp pattern Y6 support the thesis of the Northeast Asian origin for the first settlers of the American continent. For Zoubov 17, the high frequency of the protostylid in grade 1 (point P) as a unique feature of American populations that allows him to propose the existence of a Americanoid Dental Complex, consisting of all the American Paleo-Indian and the contemporary populations that are derived from them.

Regarding the Colombian population, the study of dental morphology and its association with dental complexes reviewed through ethno-historical processes that have occurred in the country is quite complicated. Rodríguez 9 states that past indigenous peoples are characterized by presenting high frequencies of *winging*, *crowding*, reduced hypocone, layered fold and P point of the protostylid, which places them closer to the Paleo-Indian sinodontes of the Mongoloid Dental Complex. However, in the case of the contemporary indigenous people, the situation varies mainly due to the miscegenation that occurred with the arrival of Northern Caucasoid human groups from Western Europe (Western Dental Complex). They populated American territory in three historical periods recognized as the discovery, conquest and colonization. These groups were characterized by a high frequency of cusp expression of the cusp of Carabelli, the X cusp pattern and the + cusp pattern. However, the dental morphology is simplified given the low frequency of shovel-shaped incisors and double-shovel pattern, the layered fold, and cusps 6 and 7 17 ^-^ 18 .

Leon and Riaño 20 analyzed a series of plaster casts of several current Colombian indigenous populations obtained by the Human Expedition conducted by the Pontificia Universidad Javeriana. The results indicate a high frequency of shovel-shaped incisors, a low frequency of the Carabelli cusp, a high proportion of hypocone reduction, variable frequency of sixth cusp, a high frequency percentage of the seventh cusp, high percentages of the upper layered fold and high frequencies of the protostylid . Cerón 21 compared some discrete dental and cranial traits among a group of Andean mestizos and a native Embera population, noting that the latter group had high frequencies of shovel-shaped incisors and cusp 6. Similarly, Negroid human groups (Southern Caucasoids from the Western Dental Complex) were brought as slaves to the South American continent and were distributed throughout different regions of Colombia. For this reason, in virtue of macro- evolutionary process represented by numerous migrations, contacts and isolations, the multiethnic, multicultural and polygenic nature of the Colombian population arose as described by Yunis *et al*., in 1992 and Ramos *et al*. in 1993 - cited by J.V. Rodríguez 9 - so that the tri-ethnic genetic composition average in Colombian remains constituted by Caucasoid genes (62%), Mongoloid (26%) and Negroid (12%), which were distributed differentially throughout differing regions of the country .

Perhaps it is in the region of Southwestern Colombia where these processes are accentuated and in which the greatest number of studies have been carried out in populations described as Caucasoid Mestizos, Indigenous and Afro-Colombian. The Research Group of Oral and Maxillofacial Surgery at the University del Valle has studied several populations from the southern part of the department (i.e. province) of Valle del Cauca and the northern part of the department of Cauca, and inferred that the frequency of TCMTs reflect historical miscegenation and dominance of these phenotypic expressions. This occurred in such a way that a group of Mestizos in the city of Cali was characterized in a simplification of the dental morphology as having a low frequency of the cusp of Carabelli (this trait was expressed ambiguously in their fossa forms - characteristic of sinodonts - and cuspids of medium size - a characteristic of Caucasoids), a reduced hypocone (a property of Western Caucasoid groups) and the high frequency of the point P of the protostylid (an exclusive feature of Amerindian populations ) 4.

Similarly, by studying two groups of Afro-Colombians from the Department of Cauca, one from the city of Puerto Tejada 22 and the other from the city Villarica 22, high frequencies of Carabelli´s cusp were observed of a medium size, groove pattern +, ¨X¨ cusp pattern, and a high frequency of cusp 7, suggesting notable influence by the Western Dental Complex. Concerning the Afro-Colombian populations, Delgado 24 indicates that the populations were derived from Africans who came to the American continent as slaves from West Africa, Central West (Sub-Saharan Africa), South-East and North, all of which are ranked in the Western Dental Complex (Southern Negroid).

The term ¨dental complex¨ refers to the characterization of large population groups, according to a specific combination of TCMTs, and since modern human groups have the same number of these features in both dentitions, the only detectable difference is in the frequencies of these traits. That is why it is necessary to cover a wider range of population groups with significant samples.

According to the results obtained from this study, it can be said that the indigenous group of Nasa who formed the sample for this study have high frequencies of TCMT characteristic of the Mongoloid Dental Complex. Specifically with the sinodonts, and similarly with other Colombian and American indigenous groups, the findings are consistent with those reported by Turner^15^, Hanihara 16, Zoubov 17 and Rodríguez 9, which also coincide with the theory of the Mongoloid origin of the indigenous tribes of South America. However, the significant frequencies of some features suggest the influence of contemporary Caucasoid human groups (Fig. 1).

## Conclusions

The most frequent TCMT in both dentitions were shovel-shaped forms, the groove shapes and fossa forms, Carabelli cusp, the metaconules, the Y6 cusp pattern, the layered fold, protostylid (point P) and the cusp 6. The TCMT observed did not display sexual dimorphism and had bilateral symmetry in their expression. The Nasa indigenous group who constituted the sample had a high affinity with ethnic groups belonging to the Mongoloid Dental Complex. This can be corroborated with the distance matrix and dendrogram obtained from the frequencies of the TCMT shovel-shaped incisors, Carabelli cusp, the protostylid, the cusp 6 and cusp 7. However, one cannot rule out the influence of Caucasoid Dental Complex associated with ethno-historical processes that occurred in Southwestern Colombia.

This research found new elements of invaluable ethnographic value from the analysis of dental morphology that eventually will allow us to understand the human diversity of this region of Southwestern Colombia, the establishment of regional linkages associated with macro-evolutionary events that occurred since the settlement of America, and the concomitant mixing between populations with Mongoloid origin (American Indians), Caucasoid (Western Europeans), and Negroid (African descent).
